# Offspring of Mothers Fed a High Fat Diet Display Hepatic Cell Cycle Inhibition and Associated Changes in Gene Expression and DNA Methylation

**DOI:** 10.1371/journal.pone.0021662

**Published:** 2011-07-11

**Authors:** Kevin J. Dudley, Deborah M. Sloboda, Kristin L. Connor, Jacques Beltrand, Mark H. Vickers

**Affiliations:** Liggins Institute and the National Research Centre for Growth and Development, University of Auckland, Auckland, New Zealand; Wellcome Trust Centre for Stem Cell Research, United Kingdom

## Abstract

The association between an adverse early life environment and increased susceptibility to later-life metabolic disorders such as obesity, type 2 diabetes and cardiovascular disease is described by the developmental origins of health and disease hypothesis. Employing a rat model of maternal high fat (MHF) nutrition, we recently reported that offspring born to MHF mothers are small at birth and develop a postnatal phenotype that closely resembles that of the human metabolic syndrome. Livers of offspring born to MHF mothers also display a fatty phenotype reflecting hepatic steatosis and characteristics of non-alcoholic fatty liver disease. In the present study we hypothesised that a MHF diet leads to altered regulation of liver development in offspring; a derangement that may be detectable during early postnatal life. Livers were collected at postnatal days 2 (P2) and 27 (P27) from male offspring of control and MHF mothers (n = 8 per group). Cell cycle dynamics, measured by flow cytometry, revealed significant G0/G1 arrest in the livers of P2 offspring born to MHF mothers, associated with an increased expression of the hepatic cell cycle inhibitor *Cdkn1a*. In P2 livers, *Cdkn1a* was hypomethylated at specific CpG dinucleotides and first exon in offspring of MHF mothers and was shown to correlate with a demonstrable increase in mRNA expression levels. These modifications at P2 preceded observable reductions in liver weight and liver∶brain weight ratio at P27, but there were no persistent changes in cell cycle dynamics or DNA methylation in MHF offspring at this time. Since *Cdkn1a* up-regulation has been associated with hepatocyte growth in pathologic states, our data may be suggestive of early hepatic dysfunction in neonates born to high fat fed mothers. It is likely that these offspring are predisposed to long-term hepatic dysfunction.

## Introduction

Epidemiological studies, large-scale clinical cohorts, and experimental animal models have shown that hormonal, metabolic and nutritional disturbances during critical periods of early development can significantly impact the propensity to develop adverse health outcomes in later life (for a review see ref [1]). In particular, studies carried out in rodents have revealed that altered maternal nutrition, including relative under- and over-nutrition, results in obesity, impaired glucose tolerance and insulin sensitivity in offspring [2], [3]. We, and others, have recently reported that offspring of high fat-fed mothers develop a phenotype similar to that of the human metabolic syndrome characterised by obesity and hyperinsulinemia [3], [4] and hepatic steatosis [5], independent of post-weaning diet. The mechanisms underpinning the development of metabolic compromise in offspring are not well understood, although it is possible that maternal obesity has functional consequences on hepatic development and function in offspring. Thus, in the present study we investigated how a maternal obesogenic environment might modify pre-pubertal hepatic development in a manner that that may impact long-term hepatic functions.

The fetal liver is a key target for altered conditions *in utero*. Developmental processes within the liver are well characterised and are known to be regulated by extracellular signals that instruct cells to proliferate, differentiate, or undergo apoptosis. Further, the liver plays a pivotal role in co-ordinating metabolic processes [6], [7] so it is likely that any environmentally induced phenotypic alterations will have long-term consequences. Indeed, a number of animal studies have revealed that maternal high fat nutrition during pregnancy and lactation leads to gross phenotypic changes in the liver of adult offspring, most notably resulting in non-alcoholic fatty liver disease (NAFLD) [5], [8]. In this study we sought to determine if a MHF diet during pregnancy and lactation leads to detectable phenotypic changes in the liver of offspring in the early postnatal developmental period and if so, by what mechanism. The cell cycle describes the process by which genetic material within a cell is replicated and segregated into two distinct membrane-bound cell compartments. This is the driving force of proliferation-mediated growth and differentiation of tissues and organs, which is an essential component of organismal development. As such, altered cell cycle dynamics, mediated by proteins that enhance or restrict progression at various cell cycle stages, can have a major impact on phenotypic outcomes. Herein we report novel observations that MHF diet during pregnancy and lactation leads to epigenetic modifications (DNA methylation) in the liver of offspring during early postnatal life that are associated with the compromised regulation of cell cycle-related genes. These changes were associated with corresponding alterations in cell cycle dynamics, and with overall reduced liver size at a later time-point. We propose that these changes may at least in part explain the increased risk of metabolic dysfunction observed in these offspring as adults.

## Results

### Maternal high fat (MHF) diet leads to inhibition of G1/S-phase cell cycle transition in the livers of male neonates at postnatal day 2 (P2)

**Figure 1:**
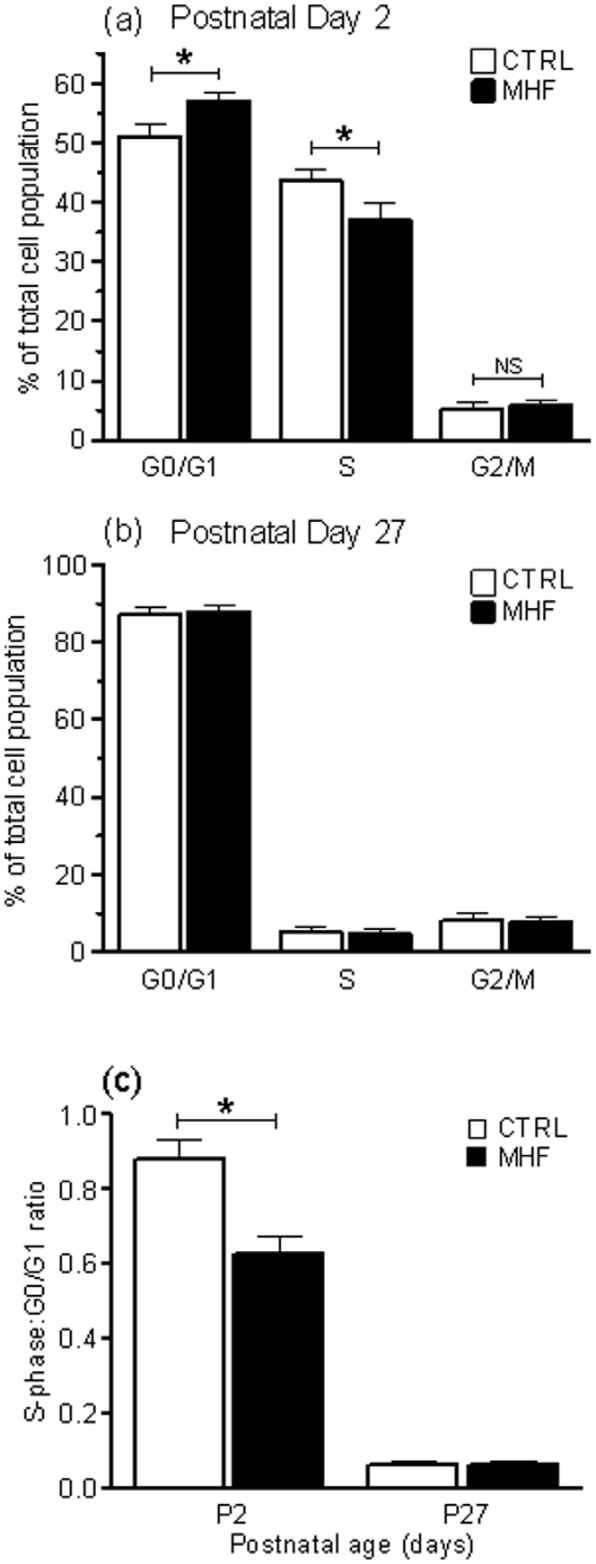
Cell cycle dynamics are altered in postnatal day 2 livers as a consequence of maternal high fat diet. (A) Graph representing the proportion of cells present in G0/G1, S and G2/M stages of the cell cycle in postnatal day 2 livers of CTRL and MHF offspring. (B) Same data representation in postnatal day 27 livers. (C) Ratio of cells in a proliferative (S) phase relative to a resting (G0/G1) phase at postnatal day 2 and postnatal day 27 in CTRL and MHF offspring. CTRL, control offspring; MHF, maternal high fat offspring; P2, postnatal day 2; P27, postnatal day 27.

**Figure 2:**
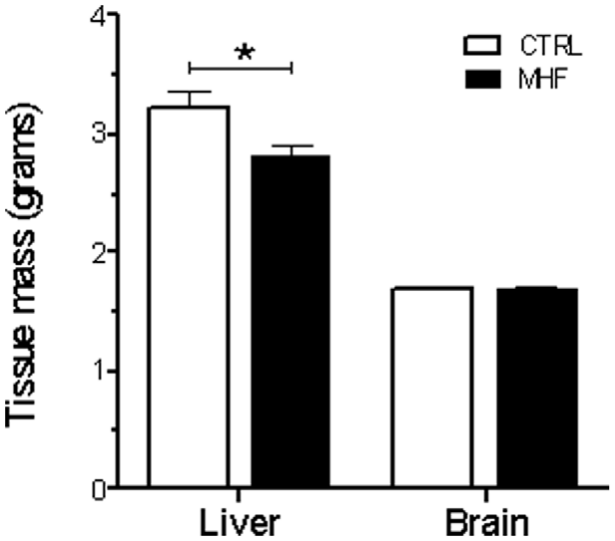
Liver weights are reduced in maternal high fat diet offspring at postnatal day 27. Liver and brain weights in CTRL and MHF offspring at postnatal day 27. CTRL, control offspring; MHF, maternal high fat offspring.

Most cells (including hepatocytes) require DNA replication to occur prior to mitosis; therefore fluorescently labelling the DNA of all cells within a population and then measuring fluorescence emitted by each individual cell (thus allowing quantification of DNA content) provides an accurate estimation of cell cycle dynamics. To determine whether differences exist in cell cycle dynamics within the liver of rats born to MHF versus CONT offspring during the first week of postnatal life (i.e. a period of active proliferation), we fluorescently labelled the DNA of dissociated liver cells from P2 rats, and measured DNA content by flow cytometric analysis. Interestingly, MHF diet was associated with a significant inhibition of transition from G_1_ to S-phase, as indicated by an increased number of low fluorescent relative to high fluorescent recordings in cell populations derived from the livers of these offspring (Figure 1a and 1c). These data are strongly suggestive of reduced cell proliferation occurring within the liver of MHF-fed offspring (compared to controls) at this time. In addition, in agreement with our previous findings, we also observed a small but significant reduction in neonatal body weight in MHF offspring at P2 (control 6.3±0.1 g, MHF 5.9±0.1 g; *p*<0.001). We previously reported that these animals display significant “catch up” growth during the first few weeks of postnatal life [3], [4], and indeed this was shown to be the case (control 61.8±1.1 g, MHF 64.8±1.1 g, *p<0.05*). As the liver is known to possess strong regenerative capabilities throughout life, we hypothesised that an increase in liver size would parallel this overall catch up growth, and to address this we investigated liver-specific effects of MHF diet in offspring at an early post-weaning time-point (i.e. P27) subsequent to catch-up growth. No differences in cell cycle dynamics were observed between CONT and MHF offspring in P27 livers, with the majority of cells appearing in a quiescent (G_0_/G_1_) phase irrespective of maternal diet (Figure 1b). Interestingly however, liver weights of MHF offspring remained significantly lower than controls at P27 (p<0.0005; Figure 2), suggesting that catch-up growth in this organ had failed to take place. These data are in stark contrast to other organs including the brain, which displayed comparable weights between control and MHF offspring at P27 (Figure 2). Overall, these results suggest that MHF diet leads to impaired proliferation-mediated growth in male offspring at a critical stage of early postnatal development, and that an inability to restore normal hepatic size during a time of general somatic catch-up growth might contribute to the long-term metabolic consequences previously reported [3], [4].

### Altered cell cycle dynamics in neonatal male livers are associated with changes in the expression of key cell cycle regulatory genes

**Figure 3:**
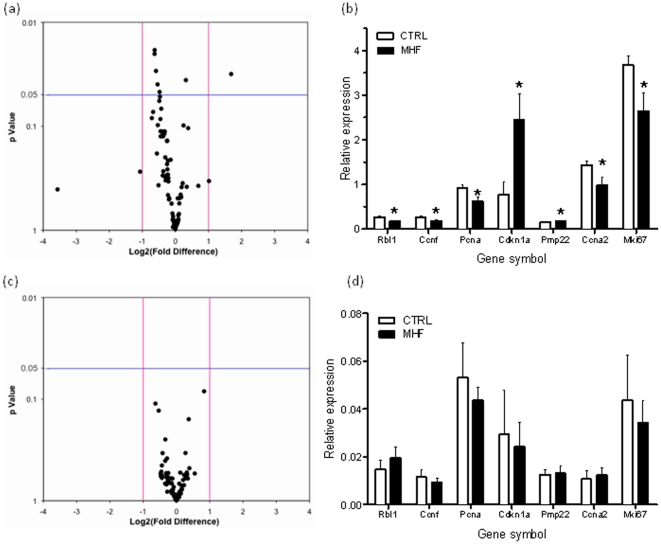
Cell cycle associated genes are differentially regulated as a consequence of maternal high fat diet. (A) Volcano plot of relative gene expression (maternal high fat offspring relative to control) for 88 cell cycle associated genes in postnatal day 2 livers. Pink vertical lines indicate +/− two fold expression threshold. Blue horizontal bar represents statistical significance (P<0.05) threshold. (B) Significantly differentially expressed genes in P2 livers (C) Volcano plot for postnatal day 27 livers (D) Relative expression profiles at postnatal day 27 for the same genes shown to be significantly differentially expressed at postnatal day 2. CTRL, control offspring; MHF, maternal high fat offspring.

We next set out to investigate the mechanism regulating altered hepatic cell cycle dynamics in P2 neonates as a consequence of MHF diet. Many genes involved in the regulation of cell cycle progression are well characterised and we suspected that altered regulation of these genes might be an important contributing factor. As such, using a validated quantitative RT-PCR array (SABiosciences) in P2 neonatal livers, we undertook differential expression analysis of 84 cell cycle-related genes. Of these, 7 genes were significantly differentially expressed between the groups (p<0.05; Figure 3a and b), the majority of which (5 out of 7) displayed reduced expression in livers from MHF neonates. Interestingly, these down-regulated genes encoded known cell cycle promoting proteins including proliferating cell nuclear antigen (PCNA), cyclin A2, and cyclin-F, whereas in contrast, *Cdkn1a*, which encodes the cell cycle inhibitory protein p21^CIP1/WAF1^ [9], [10], was significantly up-regulated in MHF neonatal livers compared to controls (Figure 3b). Similar PCR array analysis of cell cycle related genes in P27 livers revealed no significant expression differences in MHF compared to control offspring (Figure 3c and d). Taken together, these data support our hypothesis that altered cell cycle dynamics during early postnatal life are associated with impaired hepatic growth, and suggest that altered transcriptional regulation of cell cycle related genes at an important stage of neonatal liver development might be a driving factor in long-term reduced organ size. As such, these results identify a plausible mechanism by which cell cycle progression is inhibited in the livers of MHF offspring during early postnatal life.

### Maternal high fat diet leads to changes in DNA methylation in Cdkn1a

**Figure 4:**
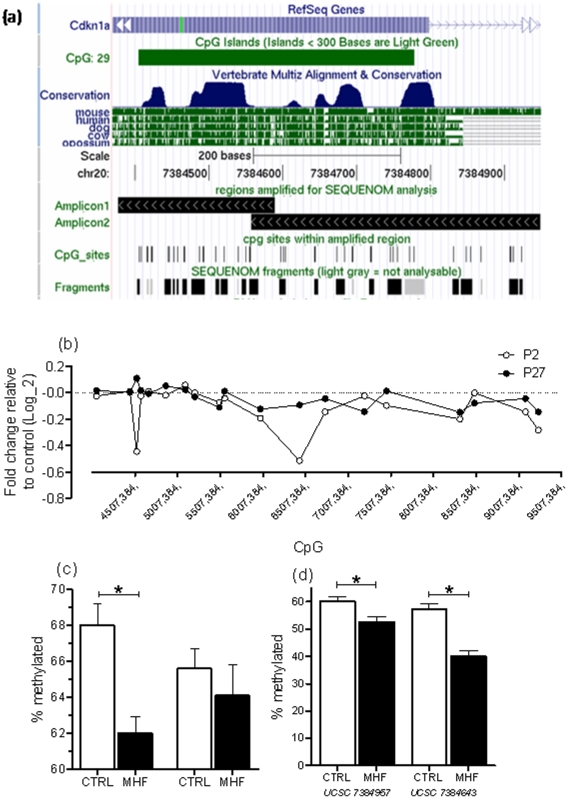
Cdkn1a DNA methylation levels are dynamically regulated in early postnatal livers. (A) UCSC browser schematic of the Cdkn1a region analysed using SEQUENOM. Amplicons (1 and 2), individual CpG dinucleotides (CpG sites) as well as the 19 analysable T-cleavage-derived CpG units (SEQUENOM fragments) are represented as custom tracks. (B) Relative difference in DNA methylation (MHF compared to control) in liver at 19 CpG units across the Cdkn1a CpG Island (UCSC position chr20:7384350–7384590) at postnatal day 2 and at postnatal day 27. (C) Average absolute DNA methylation levels in liver across the entire CpG Island in CTRL and MHF offspring at postnatal day 2 and postnatal day 27. (D) Absolute DNA methylation at specific CpG units shown to be significantly differentially methylated (positions 7384957 and 7384643) in CTRL and MHF postnatal day 2 livers. P2, postnatal day 2; P27, postnatal day 27; CTRL, control offspring; MHF, maternal high fat offspring.

**Figure 5:**
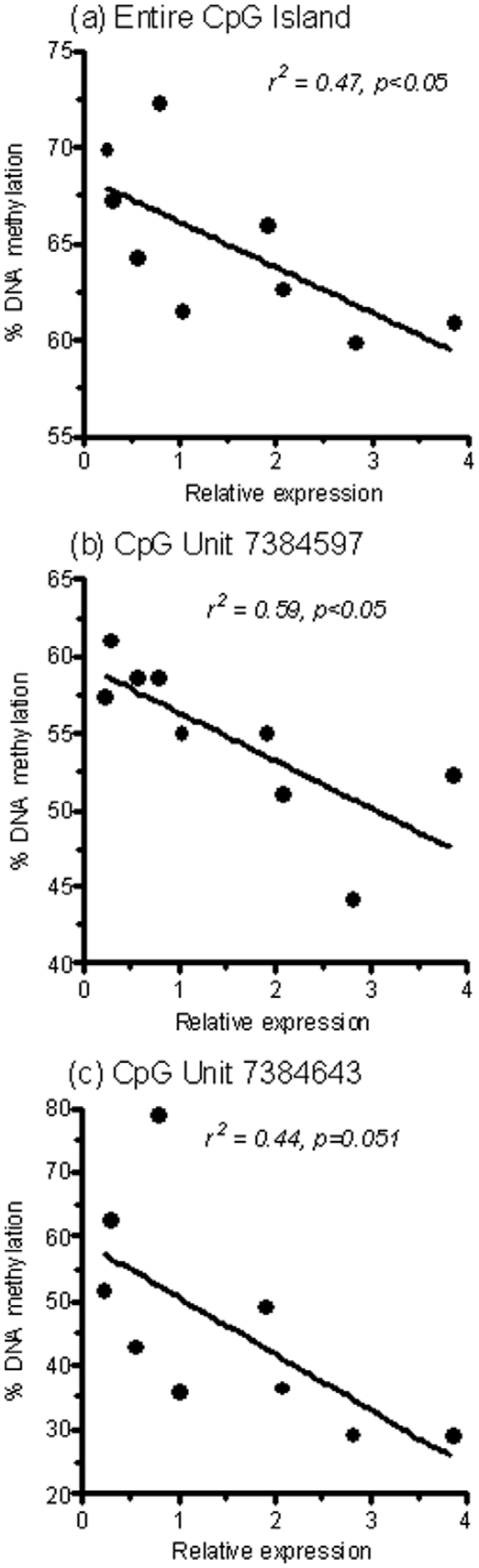
Cdkn1a DNA methylation is correlated with gene expression in postnatal day 2 livers. (A) Pearson correlation for average DNA methylation across the entire Cdkn1a CpG Island and relative gene expression in postnatal day 2 livers. (B) Correlation for DNA methylation at CpG unit 7384597 and Cdkn1a relative gene expression. (C) Correlation for DNA methylation at CpG unit 7384643 and Cdkn1a relative gene expression.

As *Cdkn1a* mRNA levels were significantly up-regulated in MHF neonatal livers, we hypothesised that alterations in DNA methylation might play a role in regulating the expression of this gene. We therefore performed quantitative DNA methylation analysis using the SEQUENOM MassArray platform. Nineteen T-cleavage restriction fragments from two PCR amplicons spanning a combined ∼600 bp region across the *Cdkn1a* CpG island (CGI) (UCSC coordinates chr20:7384350–7384590 NCBI build 39) allowed us to analyse 28 CpG dinucleotides, 12 of which at single CpG dinucleotide resolution (Figure 4a). This analysis demonstrated high levels of DNA methylation across the entire CGI in both P2 and P27 livers (Figure S1a and S1b). Interestingly, average DNA methylation across this region was significantly reduced in MHF neonatal livers relative to controls at P2 (Figure 4b and c), thus suggestive of epigenetic regulation of *Cdkn1a*. Further detailed analysis identified two CpG dinucleotides (corresponding to UCSC coordinates 7384597 and 7384643) that independently displayed significant hypomethylation relative to controls (14% and 43% hypomethylation respectively, Figure 4d). Significant correlations were observed between *Cdkn1a* gene expression and DNA methylation for the CGI as a whole and for DNA methylation at the UCSC position 7384597 CpG dinucleotide alone (Figure 5a and b). There was also a trend towards a correlation for the CpG dinucleotide at position 7384643, although this did not reach statistical significance (p = 0.051, Figure 5c). In contrast, no differences in DNA methylation status were detected in P27 livers, either for the CGI as a whole, or for individual CpG dinucleotides (Figure S1b). Based on these findings, we propose that altered DNA methylation dynamics during neonatal liver development are associated with altered regulation of *Cdkn1a* transcription, which in turn is responsible for reduced hepatic proliferation and hence liver size, in MHF offspring.

## Discussion

We previously reported that a maternal high fat (MHF) diet during pregnancy and lactation leads to changes in phenotypic outcomes resulting in obesity in later life irrespective of postnatal diet. In the present study, we report novel observations that a MHF diet during pregnancy and lactation leads to changes in epigenetic gene regulation in the neonatal liver that result in compromised regulation of cell cycle progression, changes in *Cdkn1a* gene expression and corresponding DNA methylation levels. Since *Cdkn1a* up-regulation has been associated with hepatocyte growth in pathologic states, our data may be suggestive of early hepatic dysfunction in offspring born to MHF mothers. It is likely that these offspring are predisposed to long-term hepatic dysfunction, which in addition to our previously reported obesogenic phenotype, will significantly compromise metabolic health in these offspring.

The final three days of gestation in the fetal rat are marked by a tripling of liver mass and the replacement of haematopoietic cells by hepatocytes, which is accounted for by a high rate of hepatocyte proliferation [11]. This is followed by synchronised proliferation of hepatocytes during the first week of postnatal life, prior to transition to a quiescent phenotype [12], [13]. Therefore the immediate postnatal period represents a critical stage of neonatal liver development. Our data are consistent with previous studies that have reported that maternal malnutrition leads to G_1_ cell cycle arrest in late gestation fetal rats [14]. We recently reported that MHF diet was associated with impaired placental growth at embryonic day 21 [15], consistent with maternal malnutrition models [16], [17]. This potentially explains how two contrasting maternal environments (i.e. malnutrition and high fat diet) can lead to almost identical outcomes in relation to cell cycle dynamics within the developing liver.

In contrast to many other differentiated cell types, hepatocytes are known to retain a strong propensity to replicate and rejuvenate into adulthood (for a review see [18]. This is exemplified in experimental models demonstrating almost complete tissue regeneration following two-thirds hepatectomy [18], [19]. Therefore, despite the delay in cell proliferation that inhibition of G_1_ to S-phase transition is expected to confer, we hypothesised that such differences would be reversed as a consequence of postnatal liver rejuvenation. However, this proved not to be the case, and MHF livers remained significantly smaller than controls by P27 despite somatic “catch up” growth (total body weight). It is possible that such disproportionality between liver and body weight is in itself one of the factors that contributes to the metabolic complications that arise in the offspring of MHF-fed mothers [3]. Work by Bruce *et al.* showed that a MHF diet during pregnancy and lactation resulted in “developmental priming” of non-alcoholic fatty liver disease (NAFLD) whereby maternal fat intake led to disease progression in offspring via impaired hepatic mitochondrial metabolism and up-regulated hepatic lipogenesis [5]. It is also likely that a persistent inability for liver rejuvenation throughout life might be the trigger for symptoms such as hepatic steatosis and NAFLD that have been previously reported [5], [8]. Up-regulation of p21 protein is associated with impaired regeneration of fatty livers in the *ob/ob* mouse and hepatocyte expression of p21 is increased in liver biopsies from patients with alcoholic liver disease who exhibit impaired hepatocyte proliferation [20]. Further, targeted in-vivo over-expression of p21 halts hepatocyte cell-cycle progression, postnatal liver development and regeneration in transgenic mice [21]. To test this hypothesis, it will be important for future studies to determine whether offspring of mothers fed a high fat diet display reduced propensity for liver regeneration following hepatectomy.

Epigenetic mechanisms are known to play an important role in the regulation of gene expression and developmental processes [22], [23], and have been shown to play a pivotal role in the early postnatal development of the liver [24] as well as many other developmental processes. *Cdkn1a* has previously been shown to be subject to epigenetic control in various types of cancer [25], [26], [27] and is highly methylated in cirrhotic liver [26]. Due to its known susceptibility to epigenetic regulation and potential role in obesity, *Cdkn1a* has also recently been described as an *epiobesigene* [28]. Consistent with this description, intrauterine growth restriction (IUGR) in the rat was associated with persistent epigenetic changes and corresponding changes in expression of hepatic IGF-1 [29]. Here, we have found that a MHF diet results in reduced levels of growth-promoting genes (e.g. PCNA, cyclin A) and increased expression levels of the gene encoding the cyclin-dependent kinase inhibitor p21^CIP1/WAF1^ (i.e. *Cdkn1a*) in neonatal livers. We therefore investigated epigenetic regulation of *Cdkn1a* expression in the liver of offspring, specifically focussing on DNA methylation. DNA methylation is the best-characterised mechanism of epigenetic regulation, and in most cases, high levels of DNA methylation at CpG Islands (CGI's) have been strongly correlated with transcriptional repression [30]. Our analysis revealed that increased hepatic expression of *Cdkn1a* mRNA expression in MHF offspring at P2 is associated with subtle but significantly lower levels of DNA methylation. Interestingly, these effects were seen across the entire CGI, as well as at specific CpG dinucleotides. These observations are suggestive of a mechanism by which transcriptional activity of *Cdkn1a* becomes transiently increased in P2 MHF offspring as a consequence of relatively lower DNA methylation levels. Interestingly, no differences in DNA methylation at the *Cdkn1a* CGI were observed at P27, suggesting that this epigenetic modification is dynamically regulated during early postnatal development in the rat. It will be important for future studies to determine whether differences in other epigenetic modifications such as trimethylated histone 3 lysine 27 (H3K27me3) persist at later developmental time-points. For example, previous work in cancer has shown that the polycomb modification H3K27me3 and DNA methylation are mutually exclusive and that switching between these two states can occur; thus representing a means by which epigenetic states may change during disease and development [31], [32].

High levels of DNA methylation presumably bring about their transcriptionally repressive effects by causing local chromatin compaction and thus preventing transcriptional activators from binding to gene promoter and enhancer regions. Indeed, such a phenomenon has previously been reported in the brains of rodents exposed to poor maternal care during early postnatal life [33], [34]. It is plausible that relative DNA hypomethylation at specific CpG dinucleotides, as observed in the livers of P2 rats in this study, might lead to differential transcriptional activity by modulating the binding capacity of specific transcription factors. We therefore analysed putative transcription factor binding sites across the entire *Cdkn1a* CGI region, using TRANSFAC software. This led to the identification of numerous transcription factor binding sites associated with the *Cdkn1a* CGI (see Figure S2). Of particular note was the identification of an epidermal growth factor receptor specific transcription factor (ETF) binding site associated with one of the CpG dinucleotides that were differentially methylated in P2 livers (i.e. UCSC position 7384597). Interestingly, binding of ETF to an identical site within the promoter region of another important cell cycle regulator (i.e. *TP53*) was previously shown to play an essential role in transcriptional activation [35]. In addition, it was also recently shown that ETF is involved in cytokine-independent proliferation of murine hepatocytes [36]; therefore future studies should determine whether DNA methylation at the *Cdkn1a* ETF binding site plays a critical role in the modulation of transcriptional activity, and cell proliferation, in rodent hepatocytes.

The liver is a dynamic organ comprised of many cell types, each of which possesses distinct epigenotypes. These different epigenotypes ultimately control cell functionality, and thus phenotype. In the present study we have shown that a maternal obesogenic diet is associated with changes in gene expression and DNA methylation patterns in male offspring related to cell cycle control which are paralleled by a reduced maturation of hepatocytes during a critical period of liver development. We recognise that these data are limited however, due to the presence of a mixed cell population via use of whole liver homogenates. Recent work by Grigoriu *et al.* has demonstrated significant differences in cell-type specific epigenetic regulation within a single tissue [37] and further studies are now required to target changes in specific hepatic cell populations. Nonetheless, the observation that there is a critical window for liver development during the first week of postnatal life gives plausibility to the hypothesis that epigenetic modifications caused by nutritional adversity at this time may be responsible for long-term phenotypic consequences. Future studies should concentrate on characterising these effects in further detail, and on the development of strategies to potentially reverse these effects.

## Materials and Methods

### Ethics statement

All animal work was approved by the Animal Ethics Committee at the University of Auckland (approval R712).

### Animal model

A rat model of maternal high fat nutrition was used as described previously [3], [38]. Briefly, at postnatal day 120, female Wistar rats were time-mated using an estrus cycle monitor (EC40, Fine Science Tools, CA, USA). Upon confirmation of mating, two maternal dietary groups were established: (1) Control females maintained on a standard chow diet (18% kcals from fat, Harlan Teklad Diet 2018, Harlan, Oxon, United Kingdom) throughout pregnancy and lactation and (2) females fed a high fat diet throughout pregnancy and lactation (MHF, 45% kcals from fat, D12451, Research Diets, NJ, USA). Following birth, pups were weighed and on postnatal day 2 (P2) litter size was randomly adjusted to 8 pups per litter to ensure standardized nutrition until weaning. At weaning (P22), male offspring were housed 2 per cage and fed a standard control diet *ad libitum* until postnatal day 27 (P27). Hepatic tissue samples were collected from males at 2 developmental ages, postnatal day P2 and P27 (n = 4 litters per group, *n* = 2 offspring per dam; 8 offspring per group total). Livers were weighed and a sample of liver homogenate collected in Type IV collagenase (Sigma-Aldrich) for flow cytometry and another sample from the same lobe snap frozen in liquid nitrogen for molecular analyses.

### Cell cycle analysis

A single suspension of liver-derived cells was prepared using an enzymatic dissociation procedure. Freshly excised liver was minced using a scalpel blade and approximately 200 mg was transferred to 10 ml of ice-cold HBSS solution containing 1.25 mg/ml Type IV collagenase (C5138-1G, Sigma-Aldrich). Suspensions were incubated at 37°C for 30 minutes with occasional gentle mixing by inversion, followed by triturating by gentle pipetting, and then filtering first through 100 µm and then 40 µm cell strainers (Becton Dickinson). Cells were pelleted by centrifugation at 200× g for 5 minutes at 4°C, washed twice in PBS, and resuspended in PBS to a final concentration of 1×10^6^ cells/ml. Cells were co-stained with LIVE/DEAD Fixable Green (Invitrogen) and Vybrant DyeCycle Orange (Invitrogen) dyes according to the manufacturer's instructions, and fluorescence data were collected for 20,000 viable cells using the FACSAria II flow cytometry system (Becton Dickinson). Proportions of cells in the various stages of the cell cycle were determined using FCS Express Multicycle software.

### Nucleic acid preparations

Snap frozen liver (30 mg) was homogenised using a TissueLyser (Qiagen), and total RNA and genomic DNA were co-extracted using the AllPrep DNA/RNA Mini Kit (Qiagen), according to the manufacturers recommended protocol. Extracted nucleic acids were resuspended in sterile RNase/DNase free water (Ambion), and concentrations and purity of DNA and RNA were assessed using a NanoDrop 1000 spectrophotometer (ND-1000 spectrophotometer; BioLab Ltd) using NanoDrop software (version 3.1.2).

### Quantitative differential mRNA expression analysis

Total RNA (1 µg) was used to generate cDNA using the RT^2^ First Strand Kit (SABiosciences). The differential expression (CTRL versus HF) of 84 genes key to cell cycle regulation were analysed using the Rat Cell Cycle RT^2^ Profiler PCR array (SABiosciences). Quantitative PCR was performed according to the manufacturer's recommended protocol, using the SYBR Green detector on a 7900HT Sequence Detection System (Applied Biosystems). Relative mRNA expression levels were determined by the ΔΔ-C_t_ method [39], using the average cycle threshold (C_t_) of a panel of housekeeping genes (included on the array) as the normalisation factor (SDS 2.1, Applied Biosystems).

### Quantitative DNA methylation analysis

The EZ DNA Methylation Kit (Zymo) was used to prepare sodium bisulphite treated genomic DNA, predominantly according to the manufacturer's instructions, but with the inclusion of an “Alternative Cycling Protocol” (step 1: 95°C, 30 sec; step 2: 50°C, 15 min – 20 cycles alternating between steps 1 and 2). Sodium bisulphite treated DNA was used as template to amplify two discrete regions within the CpG Island (CGI) of the rat *Cdkn1a* gene under the following PCR conditions: 2 mM MgCl_2_, 200 µM dNTP mix, 0.2 Units Taq polymerase, 400 nM forward (F) and reverse (R) primers, and 10 ng sodium bisulphite treated DNA template. Primer sequences were:

F_1_:aggaagagagGGGTTTAGGTAGATTTTGGGTAGTT; R_1_:cagtaatacgactcactatagggagaaggctACCTATTCCACACAAAAACAAAAT; F_2_:aggaagagagGAGAAAAAGGTTTGGATTATGTGAT; R_2_:cagtaatacgactcactatagggagaaggctAAACCCAAAACTACCCAAAATCTAC (lower case denotes adaptor sequences required for downstream SEQUENOM analysis). A GeneAMP PCR System 9700 (Applied Biosystems) was used to carry out the PCR under the following cycling conditions: step 1: 95°C 4 min; step 2: 95°C, 20 sec; 60°C, 30 sec; 72°C, 60 sec – 45 cycles; step 3: 72°C, min; 4°C hold. The proportion of DNA methylation at defined CpG units was then determined using the MassCLEAVE for MassARRAY procedure (SEQUENOM) according to the manufacturers recommended protocols, followed by analysis using EpiTyper 1.0 software (SEQUENOM).

### Statistical analysis

Data were analyzed using JMP 7 (SAS Institute Inc., Cary, NC, USA) for Windows. All data were first tested for normality and equal variances. Data are presented as mean ± SEM unless otherwise stated. In all cases, “litters” were considered to be individual biological replicates. At least four litters per treatment group were used, and significant differences between treatment groups were determined using Student's t-test. Correlations between gene expression and DNA methylation were analysed using the Pearson test. Level of significance was set at *p*<0.05.

## Supporting Information

Figure S1 Absolute DNA methylation levels at 19 individual CpG units across the *Cdkn1a* CpG island in CTRL and MHF offspring at postnatal day 2 (a, upper panel) and postnatal day 27 (b, lower panel). CTRL, control offspring; MHF, maternal high fat offspring. * p<0.05.
(TIF)

Figure S2 Identification of numerous transcription factor binding sites associated with the *Cdkn1a* CpG island.
(TIF)
