# Supplementary figures and images for: Offspring of Mothers Fed a High Fat Diet Display Hepatic Cell Cycle Inhibition and Associated Changes in Gene Expression and DNA Methylation
Source: PLoS One. 2011 Jul 11;6(7):e21662. doi: 10.1371/journal.pone.0021662 (PMC3133558; doi:10.1371/journal.pone.0021662)

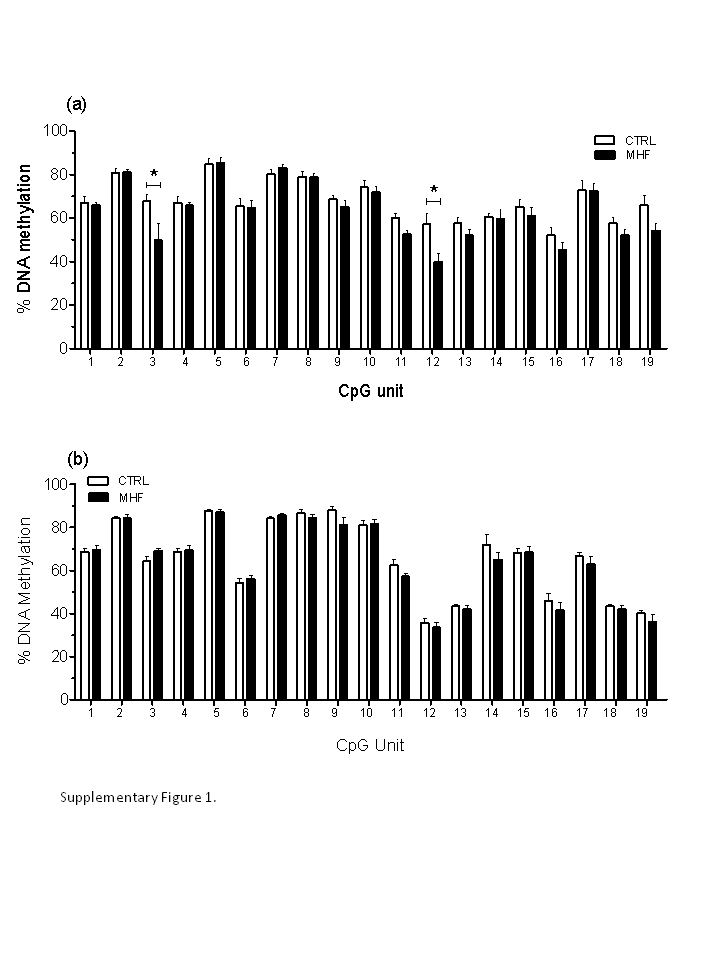

Supplement: Figure S1 — Absolute DNA methylation levels at 19 individual CpG units across the Cdkn1a CpG island in CTRL and MHF offspring at postnatal day 2 (a, upper panel) and postnatal day 27 (b, lower panel). CTRL, control offspring; MHF, maternal high fat offspring. * p<0.05. (TIF) [file pone.0021662.s001.tif]

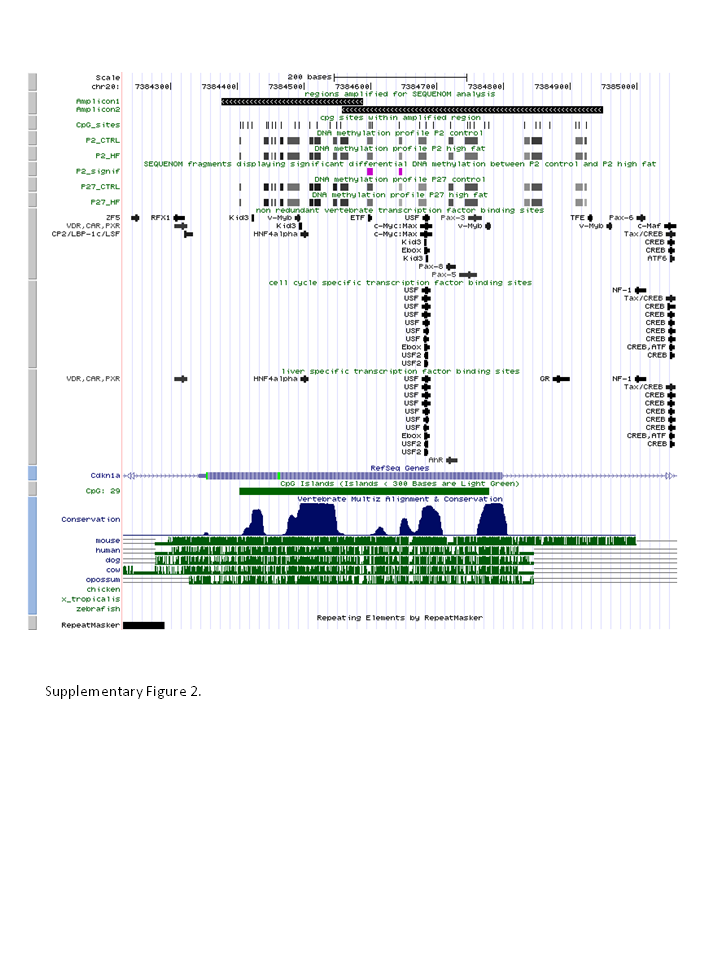

Supplement: Figure S2 — Identification of numerous transcription factor binding sites associated with the Cdkn1a CpG island. (TIF) [file pone.0021662.s002.tif]
